# Potential Role of N-Cadherin in Diagnosis and Prognosis of Diabetic Nephropathy

**DOI:** 10.3389/fendo.2022.882700

**Published:** 2022-05-31

**Authors:** Hamad Ali, Mohamed Abu-Farha, Maha M. Hammad, Sriraman Devarajan, Yousif Bahbahani, Irina Al-Khairi, Preethi Cherian, Zahra Alsairafi, Vidya Vijayan, Fahd Al-Mulla, Abdulnabi Al Attar, Jehad Abubaker

**Affiliations:** ^1^ Department of Medical Laboratory Sciences, Faculty of Allied Health Sciences, Health Sciences Center, Kuwait University, Jabriya, Kuwait; ^2^ Department of Genetics and Bioinformatics, Dasman Diabetes Institute (DDI), Dasman, Kuwait; ^3^ Department of Biochemistry and Molecular Biology, Dasman Diabetes Institute (DDI), Dasman, Kuwait; ^4^ National Dasman Diabetes Biobank, Dasman Diabetes Institute (DDI), Dasman, Kuwait; ^5^ Medical Division, Dasman Diabetes Institute (DDI), Dasman, Kuwait; ^6^ Department of Pharmacy Practice, Faculty of Pharmacy, Health Sciences Center, Kuwait University, Jabriya, Kuwait; ^7^ Diabetology Unit, Amiri Hospital, Ministry of Health, Kuwait City, Kuwait

**Keywords:** N-cadherin, diabetic nephropathy, microalbuminuria, early diagnosis, biomarker, epithelial-mesenchymal transition

## Abstract

Diabetic nephropathy (DN) is a serious complication of diabetes affecting about half the people with diabetes and the leading cause of end stage renal disease (ESRD). Albuminuria and creatinine levels are currently the classic markers for the diagnosis of DN. However, many shortcomings are arising from the use of these markers mainly because they are not specific to DN and their levels are altered by multiple non-pathological factors. Therefore, the aim of this study is to identify better markers for the accurate and early diagnosis of DN. The study was performed on 159 subjects including 42 control subjects, 50 T2D without DN and 67 T2D subjects with DN. Our data show that circulating N-cadherin levels are significantly higher in the diabetic patients who are diagnosed with DN (842.6 ± 98.6 mg/l) compared to the diabetic patients who do not have DN (470.8 ± 111.5 mg/l) and the non-diabetic control group (412.6 ± 41.8 mg/l). We also report that this increase occurs early during the developmental stages of the disease since N-cadherin levels are significantly elevated in the microalbuminuric patients when compared to the healthy control group. In addition, we show a significant correlation between N-cadherin levels and renal markers including creatinine (in serum and urine), urea and eGFR in all the diabetic patients. In conclusion, our study presents N-cadherin as a novel marker for diabetic nephropathy that can be used as a valuable prognostic and diagnostic tool to slow down or even inhibit ESRD.

## Introduction

Diabetic nephropathy (DN) is a chronic kidney disease that affects around 40% of diabetic patients of both type 1 and type 2 diabetes (1, 2). It is characterized by insistent increased levels of albumin in urine and is associated with high risk of cardiovascular morbidity and mortality (3, 4). DN is a progressive disorder where renal functions are impaired with time leading to end stage renal disease (ESRD) (5). Despite advances in the management and treatment of diabetes and associated complications, DN continues to be one of the leading causes of ESRD worldwide which highlights the need for more effective diagnostic, prognostic and therapeutic interventions (6, 7).

Clinically DN is divided into five stages; the first stage is characterized by increased glomerular filtration rate (GFR) and hypertrophy. In the second stage, microalbuminuria is noticed as a result of mesangial expansion and thickening of basement membrane leading to glomerular damage. In stage three, albuminuria increases to more than 300 mg/24h and patients do develop hypertension as well. Stage four is characterized by further elevations in albuminuria, blood urea nitrogen (BUN) and creatinine and GFR starts to decrease by approximately 10% every year. The final stage is characterized by insufficient renal functionality and ESRD (5, 8). Despite albuminuria and creatinine being the focal markers in DN diagnostic and prognostic set ups, these two markers showed a number of limitations that could obscure their clinical effectiveness (9). Several studies have shown that levels of microalbuminuria can be significantly influenced by daily physical activity, type of diet, some types of infections and hypertension which could affect its precision as a risk predictor of DN (9–14). Moreover, several diabetic patients have been shown to progress to ESRD with no elevated levels of albuminuria (15). Serum creatinine level which is widely used in monitoring and diagnosis of several chronic kidney diseases including polycystic kidney disease (16, 17) and DN is shown to be significantly influenced by many non-pathological factors including age, gender, muscle mass and hydration levels (18–21). There is a clear need for a sensitive and precise biomarker that can help in detecting DN early and in monitoring treatment outcomes.

In the past decade, efforts have been directed towards the identification of new reliable and sensitive biomarkers for the early diagnosis and monitoring of DN. Our group has identified several potential markers that are specifically elevated in DN patients when compared to diabetic controls and healthy controls. These markers include NGAL and ANGPTL4 (22). Other groups reported a different set of markers that are associated with DN pathology and renal function deterioration including urine Synaptopodin (23), urine Nephrin (23, 24), ANGPTL8 (25) and serum VEGF (26).

The Cadherins, which are a family of transmembrane proteins that are mainly involved in cells adhesion and signaling and are calcium-dependent functionally (27), have been shown to play an integral part in the establishment of renal epithelia polarization *via* their homophilic interaction through the extracellular domains of cadherins between adjacent cells (28). Among the cadherins that are expressed in the human nephron is N-Cadherin which is a 99.7 kDa protein encoded by the *CDH2* gene. This protein which is composed of 906 amino acids is normally expressed in neural tissues and cardiac muscles in addition to renal tissues (29). In acute kidney injury (AKI) due to ischemia, it has been shown that N-Cadherin is depleted from the proximal tubules in the kidney unlike E-cadherin which highlights a potential role of N-Cadherin in kidney functionality (30). From the prospective of DN, there are no solid reports on the role of N-Cadherin in DN or its diagnostic potential. On the other hand, few clinical studies showed serum and urinary elevated levels of E-cadherin in DN patients (31, 32). As N-cadherin expression has been shown to be influenced by pathological impact (AKI) where it is depleted from renal epithelial cells resulting in disruption of cellular functional organization, it might be holding a potential diagnostic benefit for DN.

In this study, we aim to analyze circulatory concentrations of N-cadherin in diabetic nephropathy patients and compare it to the levels in non-diabetic and diabetic controls to determine whether N-cadherin can serve as a biomarker for diabetic nephropathy diagnosis.

## Materials and Methods

### Subjects Recruitments

Our cohort consisting of 67 DN patients, 50 T2D with no DN and 42 healthy controls was clinically identified and recruited in Dasman Diabetes Institute (DDI). Subjects provided written informed consents before their enrollment in the study. The Ethical Review Committee (ERC) of Dasman Diabetes Institute reviewed and approved the study and methodologies in accordance with the ethical declaration of Helsinki. Participants in all groups were age and BMI matched.

### Inclusion and Exclusion Criteria

Subjects were enrolled in three groups; healthy subjects with no symptoms or history of T2D or DN; T2D subjects with no DN and DN subjects. DN patients were identified based on elevated Albumin-to-creatinine ratio (ACR) in a spot urine sample in accordance with the American Diabetes Association criteria (33). Patients’ exclusion criteria included: 1) Non-diabetic kidney disease, 2) Chronic liver disease, 3) Heart failure, 4) Current/recent infection, 5) Acute/chronic inflammatory disease, 6) Allergic condition, 7) Autoimmune disease, 8) Malignancy, 9) Patients with T1D and 10) ESRD (34).

### Sample Collection

Subjects were fasting overnight before the blood and urine samples were collected in the clinics of Dasman Diabetes Institute. Urine first-void samples were collected in 120 ml urine collection containers and blood samples were collected in EDTA tubes. Plasma was isolated from blood samples after centrifugation and then aliquoted and stored at -80°C until analysis.

### Anthropometric and Biochemical Measurements

The anthropometric data were obtained and documented. Blood pressure readings were obtained using Omron HEM-907XL Digital sphygmomanometer. Fasting blood Glucose (FBG), triglyceride (TG), total cholesterol (TC), low density lipoprotein (LDL) and high-density lipoprotein (HDL) were determined using Siemens Dimension RXL chemistry analyzer (Diamond Diagnostics, Holliston, MA). CLINITEK Novus Automated Urine Chemistry Analyzer (Siemens Healthineers, Erlangen, Germany) was utilized to determine levels of urinary albumin “spot” (Alb) and creatinine (Cr) in addition to urinary albumin–to–creatinine ratio (UACR). VITROS 250 automatic analyzer was utilized to perform fully automated Renal Function Test (RFT) and estimated glomerular filtration rate (eGFR) was calculated by the MDRD (Modification of Diet in Renal Disease) Study equation (35).

### Determining N-Cadherin Concentration Using BioPlex 200-Luminex Analysis

Thawed plasma samples were utilized to determine N-Cadherin levels in all enrolled subjects. Samples were subject to centrifugation at 10000xg for 5 minutes at 4°C to eliminate any debris. N-Cadherin concentration was determined *via* a customized 11-plex multiplexing analysis kit (R&D systems, Minneapolis, MN, USA. Cat # LXSAHM) following manufacturer protocol. A standard protein mix, provided with the kit, was used to prepare a serial dilution. The results were obtained using the BioPlex 200-Luminex system (Bio-Rad, CA, USA). Analysis of the results were done using the BioPlex manager software. No significant cross reactivity with different proteins was observed. The confidence level between the expected and the observed standard concentration levels for N-Cadherin was between 95-105% as assessed by the system. The concentrations of the N-Cadherin in the study samples were calculated using a 5-Pt standard curve.

### Statistical Analysis

Statistical analysis was performed using IBM SPSS Statistics for Windows, Version 25.0. Armonk, NY: IBM Corp. Descriptive statistics were obtained for all variables measured and then presented as (Mean ± SD). Comparisons between the groups (DN, T2D and Heathy subjects) for all the tested variables were performed using one-way analysis of variance (ANOVA). Bonferroni post-hoc was utilized to test pairwise differences. Spearman’s correlations were utilized to calculate the univariate association between N-cadherin and the other variables. A P value < 0.05 was considered statistically significant for all tests performed. The ANOVA F score was validated using the F critical table (degrees of freedom; numerator 2; denominator 156). F value above F critical and P value < 0.05 are considered to reject the null hypothesis in our population.

## Results

### Cohort Demographics

Total of 159 subjects were enrolled in this study including 42 control subjects, 50 T2D without DN and 67 T2D subjects with DN. The demographic data of the study population is shown in **Table 1**. The three groups were age and BMI matched (p-value >0.05).

**Table 1:** Demographic data of study population.

	Healthy group (n = 42)	T2D group (n = 50)	DN group (n = 67)
Gender n (%)	Males 18 (43%)	Males 22 (44%)	Males 28 (42%)
	Females 24 (57%)	Females 28 (56%)	Females 39 (58%)
Age (years) ± S.D	57.74 ± 4.55	58.96 ± 7.22	59.09 ± 5.29
BMI (kg/m^2^) ± S.D	33.20 ± 5.47	33.94 ± 6.25	34.23 ± 6.84

### Clinical and Biochemical Characteristics of Enrolled Groups

The T2D and DN groups were clinically evaluated and compared to the healthy group. Systolic and diastolic blood pressures between the three groups showed no statistical significance. Fasting glucose levels showed significant differences between the three groups (p<0.001 ANOVA) and the highest values recorded in T2D and DN groups. HbA1C was higher in T2D and DN groups when compared to the healthy group (p<0.05 ANOVA).

To assess renal functions clinical RFT was performed for all subjects. Serum Creatinine and BUN were all elevated in DN group when compared to the healthy and T2D groups (p<0.00005 for all, ANOVA). Consequently, eGFR in DN was the lowest when compared to T2D and healthy groups (p<0.00005 ANOVA). Similarly, urine creatinine was low in DN when compared with the other two groups (p<0.05 ANOVA). ACR and Microalbumin were significantly increased in DN group while the levels in the other two groups were in the normal range (p<0.005, ANOVA, for both). *Post Hoc* Bonferroni indicated significant differences between the DN group and the other two groups for all the RFT markers including the microalbumin and ACR.

### N-Cadherin Expression Level and Correlation With Renal Markers

**Figure 1:**
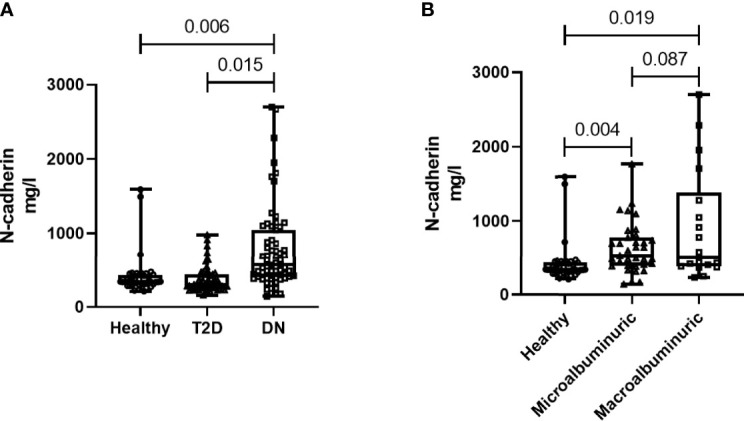
N-cadherin levels in the participants. **(A)** Based on the diabetes status. **(B)** Based on the albuminuria levels.

N-cadherin showed significantly elevated levels in the DN group when compared to the other two groups (p<0.01 ANOVA). *Post Hoc* Bonferroni indicated significant differences in N-cadherin between the DN group and the other two groups (**Figure 1A**) and **Table 2**. Reported N-cadherin results have F values greater than F critical. Furthermore, the diabetic nephropathy group was subdivided into two groups according to their Albumin-to-creatinine ratio (ACR) and was compared with the healthy group. Subjects with ACR in the range between 30 to 300 mg/g were considered microalbuminuric and subjects with ACR > 300 mg/g were considered macroalbuminuric. N-cadherin levels were significantly elevated in both groups (i.e. microalbuminuric and macroalbuminuric) when compared to the N-cadherin levels in the healthy group (**Figure 1B**). Furthermore, a trend for increase was detected in the macroalbuminuric when compared to the microalbuminuric but was not statistically significant (p = 0.087) (**Figure 1B**).

**Table 2:** Clinical and Biochemistry characteristics of study cohort.

Marker	Healthy group (± SEM)	T2D group ( ± SEM)	DN group ( ± SEM)	ANOVA (p value)	F value	*Post Hoc* Bonferroni P value adjusted for multiple comparisons with *post hoc* Bonferroni		
						T2D vs. DN	T2D vs. Healthy	DN vs. Healthy
SBP (mmHg)	122.50 ± 2.25	132.98 ± 3.88	132.03 ± 3.41	0.087	2.477	1.000	0.139	0.162
DBP (mmHg)	73.76 ± 1.52	69.72 ± 2.26	68.78 ± 1.98	0.21	1.558	1.000	0.574	0.261
Fasting Glucose (mmol/l)	5.52 ± 0.12	8.27 ± 0.36	9.61 ± 0.48	<0.001	24.763	.050	.000	.000
HbA1C (%)	5.66 ± 0.09	9.53 ± 1.73	8.09 ± 0.22	0.031	3.549	.816	.027	.236
Serum Creatinine (umol/l)	75.69 ± 2.89	79.42 ± 3.54	118.36 ± 6.57	<0.001	21.277	0.0001	1.000	0.0001
BUN (mmol/l)	5.02 ± 0.20	5.10 ± 0.29	7.53 ± 0.52	<0.001	12.338	0.001	1.000	0.0001
eGFR MDRD (mL/min/1.73 m^2^)	81.07 ± 2.14	79.22 ± 3.19	59.7 ± 3.00	<0.001	17.422	0.0001	1.000	0.0001
Albumin (g/l)	40.5 ± 0.52	37.94 ± 0.50	37.28 ± 0.42	<0.001	11.771	0.927	0.002	0.0001
Urine Creatinine (mg/l)	14.75 ± 1.22	11.94 ± 0.86	9.08 ± 0.77	0.049	9.415	0.071	0.140	0.001
Microalbumin (mg/l)	14.82 ± 2.0	14.35 ± 1.61	490.72 ± 186.62	<0.001	11.400	0.003	1.000	0.005
ACR (mg/g)	9.77 ± 1.2	11.32 ± 1.07	953.48 ± 327.	0.004	5.658	0.013	1.000	0.02
N-cadherin (mg/l)	412.56 ± 41.78	470.76 ± 111.48	842.57 ± 98.61	<0.01	6.388	0.015	1.000	0.006

Spearman’s rank correlation analysis showed significant positive correlation of N-Cadherin in one hand and serum creatinine and BUN on the other hand in DN and T2D groups. Significant negative correlations were also found between N-Cadherin and eGFR in the DN and T2D groups (**Table 3**).

**Table 3:** Spearman’s rank correlation coefficient between N-cadherin and renal markers.

	HEALTHY		T2D		DN	
	Spearman	p value	Spearman	p value	Spearman	p value
Serum Creatinine	0.094	0.556	**0.625**	**0.0001**	**0.604**	**0.0001**
BUN (blood urea nitrogen)	0.058	0.714	**0.432**	**0.002**	**0.548**	**0.0001**
eGFR (MDRD)	-0.198	0.208	**-0.647**	**0.0001**	**-0.657**	**0.0001**
Albumin	-0.101	0.525	-0.247	0.084	-0.193	0.127
Urine Creatinine	0.130	0.412	-0.091	0.531	**-0.309**	**0.013**
Microalbumin	0.208	0.187	0.046	0.750	0.019	0.879

BUN, blood urea nitrogen; eGFR, estimated GFR; (calculated using MDRD equation Modification of Diet in Renal Disease).

Bold values indicate significant correlation (P value < 0.05).

### ROC Curve Analysis of N-Cadherin

**Figure 2:**
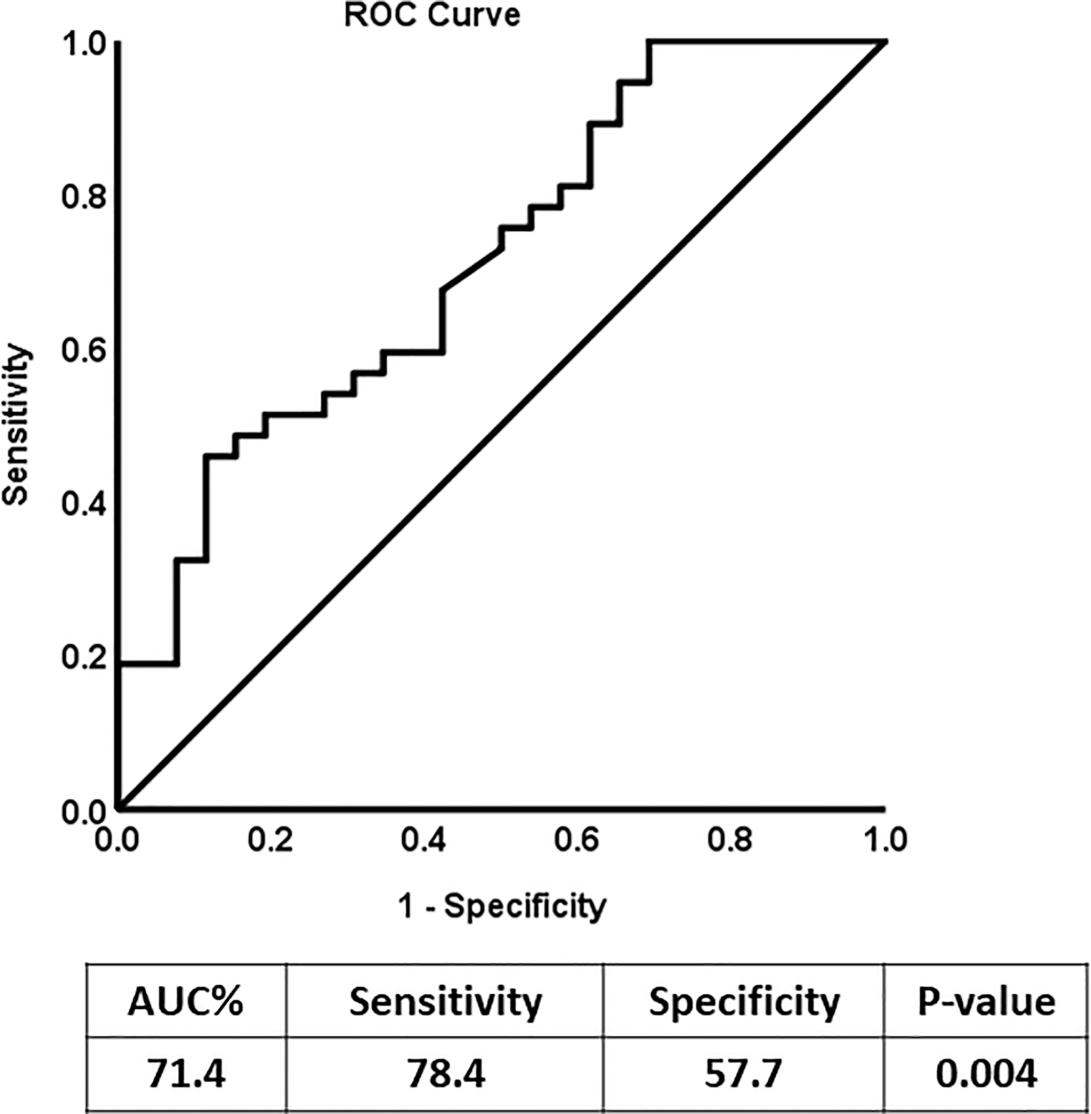
Receiver Operating Characteristic (ROC) curve analysis for N-cadherin of DN Group compared to the non-diabetic control group.

Receiver Operating Characteristic (ROC) analysis was performed to determine the possible use of N-cadherin as a marker to distinguish between diabetic patients with or without kidney complications, specifically diabetic nephropathy. The area under the curve (AUC) for the analysis was 0.76 (**Figure 2**) reflecting good ability of N-cadherin as a marker. The ROC analysis results were interpreted as follows: AUC <0.70, low diagnostic accuracy; AUC in the range of 0.70–0.90, moderate diagnostic accuracy; and AUC ≥0.90, high diagnostic accuracy.

## Discussion

This study analyzed the circulating levels of N-cadherin in diabetic nephropathy in an attempt to find sensitive prognostic and diagnostic markers for this concerning complication. Our findings show that circulating N-cadherin levels are significantly elevated in patients with DN compared to people with T2D and healthy controls. We also report that this increase occurs early during the developmental stages of the disease (second stage) as the increase was significant in microalbuminuric patients. We also show a significant correlation between N-cadherin levels and renal markers including creatinine (in serum and urine), urea and eGFR in all of the diabetic patients.

In the IDF Diabetes Atlas Ninth edition of 2019, the projected prevalence of diabetes was estimated to rise to 700 million by 2045 (36). Diabetic kidney disease (DKD) continues to be one of the major complications of diabetes affecting nearly 40% of all (type I and type II) diabetic patients worldwide (37). Therefore, with such rates, it is expected that DKD will reach epidemic scale by 2045 affecting more than 300 million people. There is currently a need for new predictive and diagnostic markers for DN given that the currently used markers (albuminuria and creatinine) are not ideal due to sensitivity and specificity issues. The treatment options are also restricted to inhibitors of the renin-angiotensin system as well as maintaining the diabetes under control by managing hyperglycemia, blood pressure and dyslipidemia. Late diagnosis and the lack of treatment options for advanced stages may cause ESRD and eventually a patient would require renal replacement therapy or a kidney transplant. Therefore, our aim from this study was to identify novel markers that could help in early diagnosis of DN. It would be of great benefit for the success of the treatment if DN is detected in the early stages.

Given the established effect of diabetes on kidney function, it is essential to perform a comprehensive evaluation on the kidneys including the different segments of the tubules. While the evaluation of GFR and albuminuria levels is a good indication for the integrity of the filtration membrane and the glomerular function, cadherin levels can more specifically reflect the function of the renal tubules. Therefore, we decided to assess the levels of N-cadherin in patients with diabetic nephropathy.

There have been several previous reports associating E-cadherin with DN (31, 32). Interestingly, serum E-cadherin levels were shown to be reduced in DN in these studies whereas we are demonstrating an increase in N-cadherin levels with DN. This indicates that different cadherin subtypes can have differential expression patterns in disease states. Such variation could be due to their distinct distribution and hence function in the kidneys. Human studies showed that N-cadherin is exclusively expressed in the proximal tubule while E-cadherin is abundant in the distal tubule and collecting duct (38, 39).

Moreover, a recent study confirmed and validated the use of urine E-cadherin as a marker for early detection of kidney injury in diabetic patients in a longitudinal setting (40). Their findings indicated not only that E-cadherin levels are elevated in nephropathy patients, but can also differentiate between the different stages of DN. They even reported that the elevation in urinary E-cadherin levels was detected 20 ± 12.5 months before the onset of microalbuminuria. Studies on E-cadherin showed some discrepancy when the tissue expression levels were measured. In one study, Jiang et al. showed E-cadherin expression in the renal tubular epithelial cells to be downregulated in DN patients compared to the healthy controls (31). In another report, Koziolek et al. analyzed kidney tissues from two biopsy cohorts. They reported a significant upregulation in DN patients in their smaller cohort of 22 samples but no difference in their larger cohort which included 201 biopsies belonging to different diseases groups (40).

To our knowledge, this is the first report to specifically correlate N-cadherin levels with renal markers in diabetic nephropathy patients. By performing Spearman’s correlation analysis, we found that there was a significant positive correlation between the levels of N-cadherin and serum creatinine as well as blood urea nitrogen. On the other hand, there was a significant negative correlation between the levels of N-cadherin and urine creatinine and eGFR. It is expected that the renal tubular epithelial cells would undergo ischemia and apoptosis during the progress of renal impairment in DN due to the inflammatory state as well as the high glucose concentrations in diabetes (41). Our correlation data suggest that N-cadherin plays an important role in renal function and its elevation could be a defense mechanism to counteract the damage and apoptosis resulting from renal injury in diabetes. Interestingly, we saw a significant increase in N-cadherin levels in DN patients who showed microalbuminuria compared to the healthy controls. This is a strong evidence that N-cadherin levels start increasing during the early stages of DN and can hence be used as an accurate diagnostic marker. The ROC analysis further supports this conclusion. We also illustrate that N-cadherin levels can be a reflection for the degree of albuminuria in DN since there was a trend of increase in the macroalbuminuric patients compared to their microalbuminuric counterparts. The lack of significance between these two groups in **Figure 1B** (p=0.087) could be attributed to the wide variation of N-cadherin levels in the participants with macroalbuminuria. Surprisingly, N-cadherin levels were reported to be reduced in response to ischemia in acute kidney injury (30), which reflects the differential role for N-cadherin in different renal diseases and that it is a disease-dependent marker.

One of the mechanisms that have been described to contribute to kidney fibrosis after injury is Epithelial-Mesenchymal Transition (EMT) (42). EMT can be defined as the conversion of differentiated epithelial cells to myofibroblasts. EMT was originally associated with cancer metastasis, however, it was later shown to play an important role in renal and tubular interstitial fibrosis. EMT entails the loss of epithelial cell adhesion leading to actin reorganization and disruption of tubular basement membrane. Interestingly, one of the consequences of EMT that was described in cancer is a phenomena called “cadherin switching” where E-cadherin levels are downregulated and N-cadherin levels are upregulated (43). When E-cadherin levels are reduced, the cell-cell junctions that are mediated by E-cadherin (including tight junctions, gap junctions and adherents junctions) dissociate and this consequently results in the stabilization of the N-cadherin-mediated junctions. Altogether, our results from this study on N-cadherin combined with previous reports on E-cadherin provide evidence that EMT and cadherin switching can occur in diabetic nephropathy and may be mediating the pathophysiological changes associated with it.

One of the main limitations of the study is its cross-sectional design hindering us from concluding causality. Therefore, additional studies are still required looking into the integrity of the tubular segments of the kidney and the components of these segments and how they can influence DN and hence be used as early detection markers. It would be interesting to measure N-cadherin levels in the context of other kidney diseases to determine if it is specific to diabetic nephropathy especially that one of the major shortcomings of serum creatinine is its common use for several kidney diseases. Furthermore, measuring the N-cadherin levels in a larger population would help in determining its clinical value as a DN predictive marker. Validation in a larger cohort using a longitudinal design is the logical next step.

In conclusion, the current study identifies N-cadherin as a novel marker for diabetic nephropathy that can be used to determine the stage of DN. Therefore, assessing N-cadherin levels in diabetic patients could have a valuable impact on slowing down or even inhibiting ESRD.

## Data Availability Statement

The original contributions presented in the study are included in the article/supplementary material. Further inquiries can be directed to the corresponding authors.

## Ethics Statement

The studies involving human participants were reviewed and approved by The Ethical Review Committee (ERC) of Dasman Diabetes Institute. The patients/participants provided their written informed consent to participate in this study.

## Author Contributions

HA: conceptualization and study design, initial manuscript drafting and revision. MA-F: data analysis and interpretation and critical revision of the manuscript. MH: data analysis and manuscript drafting and revision. SD: data analysis, management and statistical analysis. YB: patients’ recruitment and data interpretation. IA-K: performed BioPlex assay. PC: performed BioPlex assay. ZA: patient coordination and sample collection. VV: Blood processing, storage and data analysis. FA-M: Data interpretation and critical revision of the manuscript. AA: study design, data interpretation and management. JA: study design, data interpretation, wrote and critically revised the manuscript. All authors have seen and approved the final manuscript. All authors contributed to the article and approved the submitted version.

## Funding

This work was supported by the Kuwait Foundation for the Advancement of Sciences (KFAS) under projects (RA-2015-012), (PR17-13MM-07) and (RA HM 2019–008).

## Conflict of Interest

The authors declare that the research was conducted in the absence of any commercial or financial relationships that could be construed as a potential conflict of interest.

## Publisher’s Note

All claims expressed in this article are solely those of the authors and do not necessarily represent those of their affiliated organizations, or those of the publisher, the editors and the reviewers. Any product that may be evaluated in this article, or claim that may be made by its manufacturer, is not guaranteed or endorsed by the publisher.
